# Supplementary material for: Identifying SARS-CoV-2 antiviral compounds by screening for small molecule inhibitors of nsp14/nsp10 exoribonuclease
Source: Biochem J. 2021 Jul 2;478(13):2445–64. doi: 10.1042/BCJ20210198 (PMC8286829; doi:10.1042/BCJ20210198)
Supplement: Supplementary Figures S1-S5 and Tables S1-S4 [file BCJ-478-2445-s1.pdf]

Supplementary Figure Legends

Supplementary Figure S1. Titration of RiboGreen

RiboGreen was diluted as indicated and then incubated with 4 to 64 nM unlabelled double-stranded RNA (dsRNA) substrate prior to fluorescence detection. Graph shows values of a single experiment. (a.u. = arbitrary units).

Supplementary Figure S2. The nsp10-14 fusion functions as a methyltransferase

The nsp10-14 fusion protein was assayed for methyltransferase activity by the detection of formed SAH following methyltransferase assay (see Experimental Procedures). The methyltransferase reaction was run in either the absence of 10 nM nsp10-14, 1 µM SAM methyl donor, 0.11 mM GpppA cap analogue, or in the presence of all three components. In addition, the methyltransferase reaction was conducted in the presence of 2.5 µM and 25 µM of the pan-methyltransferase inhibitor Sinefungin, which acts as a competitive inhibitor (with respect to SAM) towards SAM-dependent methyltransferases. Error bars represent standard deviation from the mean of triplicate values of a single experiment. (HTRF = Homogenous Time Resolved Fluorescence).

Supplementary Figure S3. Ribogreen-based screen to identify SARS-CoV-2 nsp14/nsp10 exoribonuclease inhibitors.

A. Determination of initial rate of reaction when varying unlabelled double-stranded RNA (dsRNA) substrate concentration (15 - 250 nM). Reactions were carried out using 5 nM nsp10-14 fusion enzyme and stopped after 0, 3 and 6 minutes before dsRNA detection with RiboGreen. Graph shows values of a single experiment. **B.** Non-linear Michaelis-Menten fit for enzyme kinetics data generated by substrate titration from A. **C.** Normalised residual activity for screen samples at low and high concentrations. **D.** Selected compounds were assayed for autofluorescence at 525 nm (RiboGreen wavelength). Error bars represent standard deviation from the mean of triplicate values from a single experiment. (a.u. = arbitrary units).

Supplementary Figure S4. Patulin and aurintricarboxylic acid (ATA) inhibit nsp14/nsp10 nuclease in vitro.

A. 30 nM of co-expressed Ec F-nsp14/nsp10 complex was pre-incubated with 3 - 200 µM of patulin, and nuclease reactions were performed in the presence of 50 nM Cy5-dsRNA substrate at RT for 5 min and visualised in TBE-urea polyacrylamide gels. No (-) enzyme control was added as reference of the uncleaved substrate. **B.** Nuclease reactions containing titration of RNase A or benzonase and 50 nM Cy5-dsRNA substrate were performed at RT for 5 minutes and visualised by denaturing TBE-urea polyacrylamide gels. No (-) enzyme control was added as reference of the uncleaved substrate. **C, D.**

Representation of the chemical structures of patulin (C) and ATA (D). **E, F.** Kinetic nuclease reactions (20 min) were performed in triplicates in the presence of 0.5 nM nsp10-14 fusion, 50 nM Cy3-dsRNA substrate and 0 to 160 µM patulin (C) or ATA (D). Patulin and ATA were pre-incubated with nsp10-14 fusion for 10 minutes prior to the addition of the substrate. These reactions were used to calculate the IC_{50} of the inhibitors (see Experimental Procedures). Error bars represent standard deviation from the mean of triplicate values of a single experiment. **G, H.** Drug quenching test performed by assessing fluorescence of a Cy3-labelled oligonucleotide (50 nM) pre-incubated 10 minutes in the presence of a titration (0 - 160 µM) of patulin and ATA respectively. Graph represents values of a single experiment.

Supplementary Figure S5. Patulin and remdesivir are not synergistic to inhibit SARS-CoV-2 viral growth in VERO cells.

SARS-CoV-2 infectivity assays in VERO E6 assay performed as in Figure 5A with the indicated concentrations of patulin and remdesivir. For each condition, quantifications represent viral area normalised to the no drug well. Error bars represent standard deviation from the mean of triplicate values of a single experiment.

Supplementary Figure S1

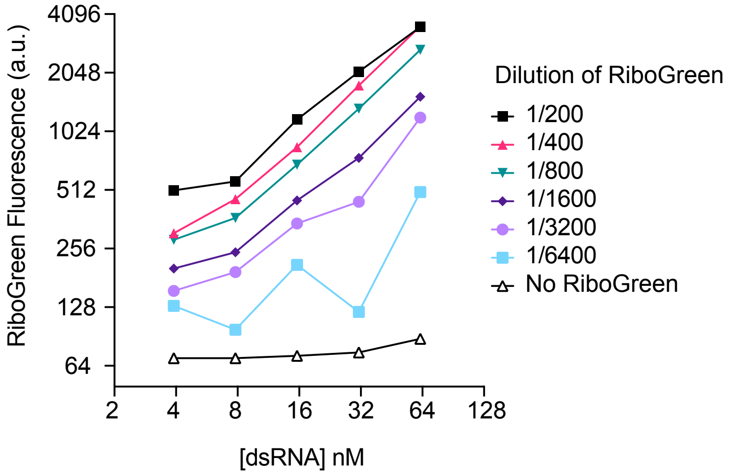

Supplementary Figure S2

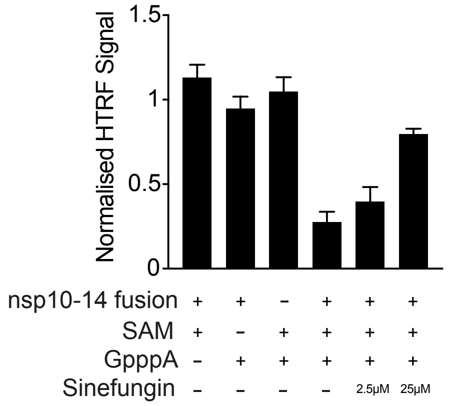

Supplementary Figure S3

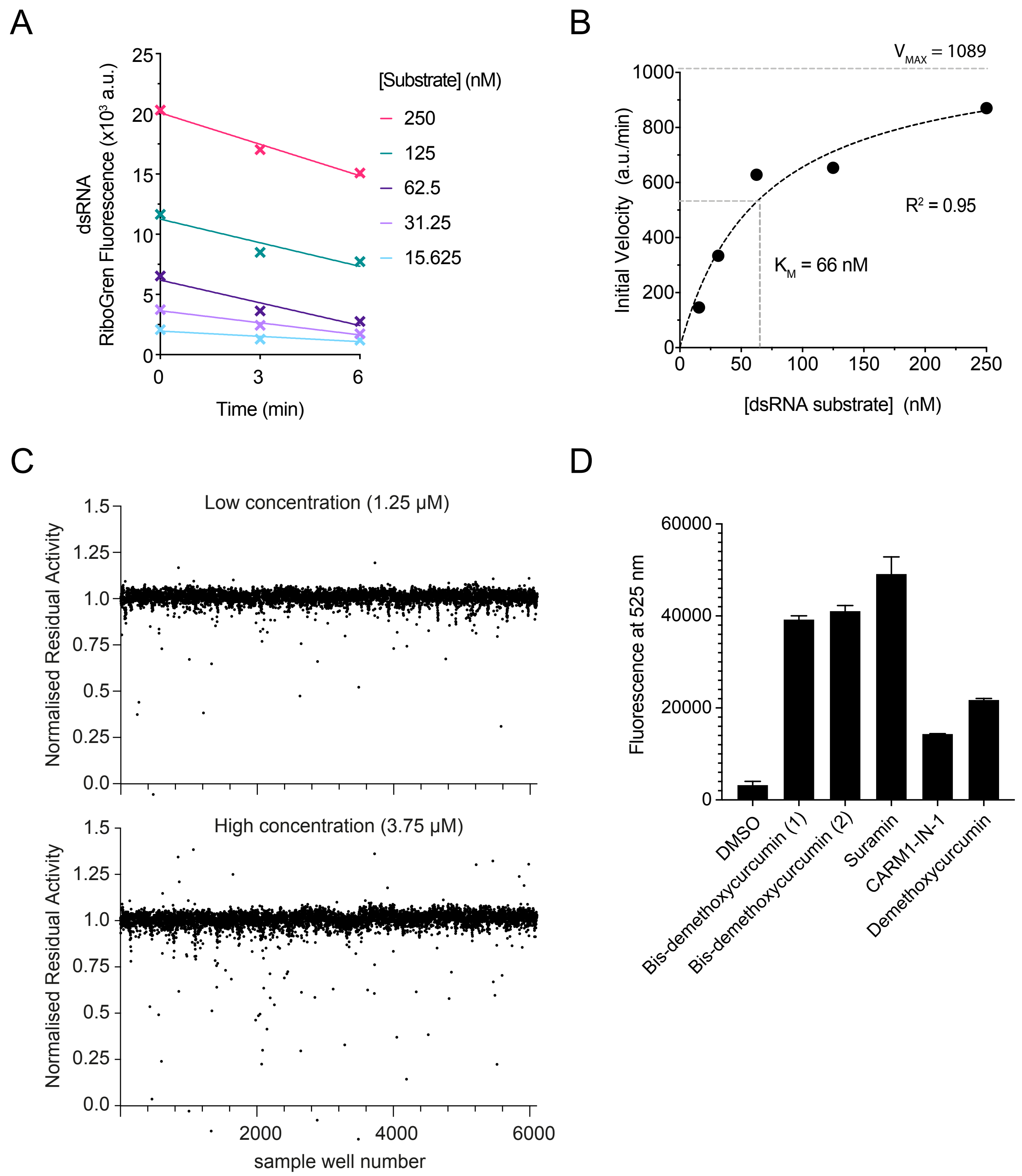

Supplementary Figure S4

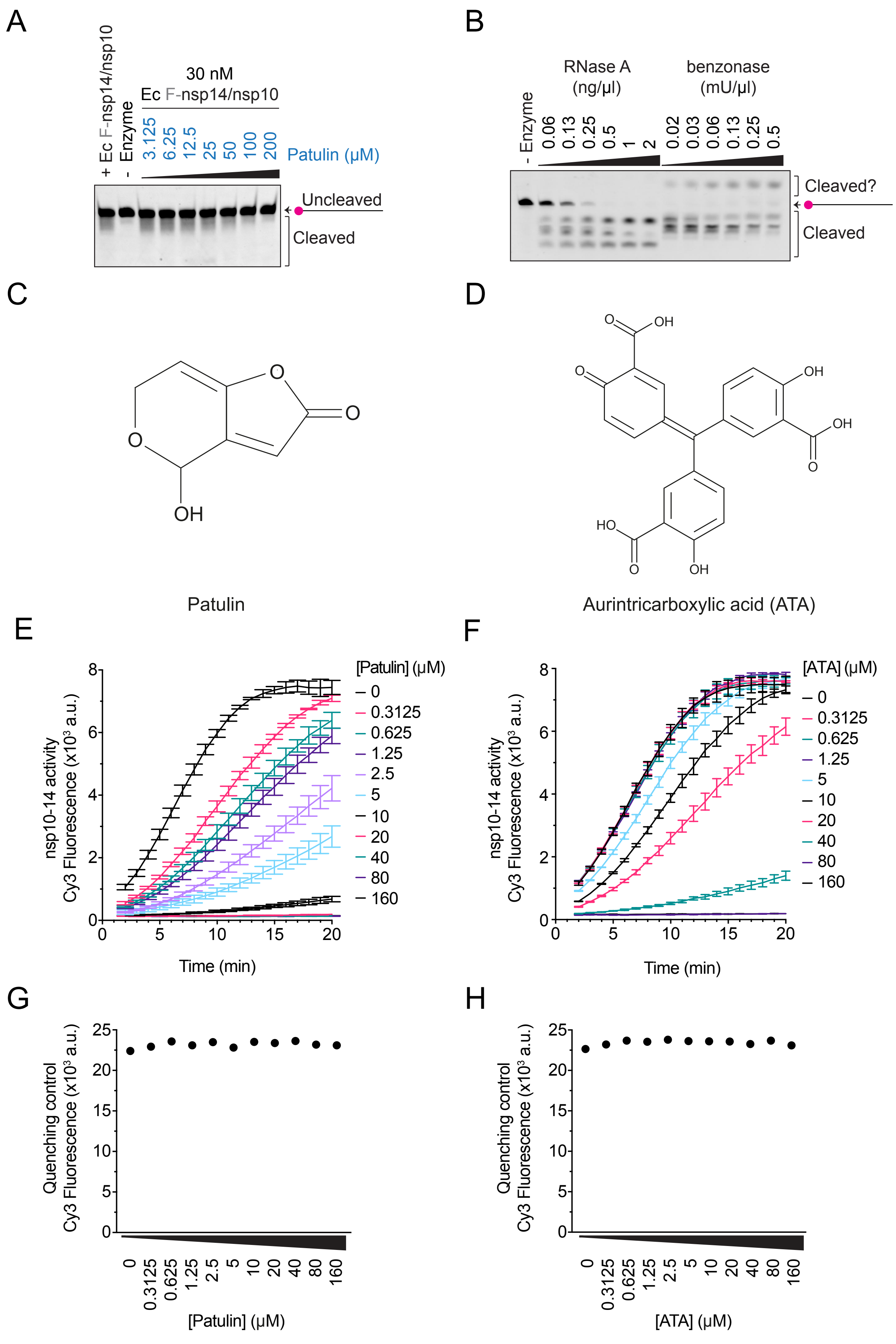

Supplementary Figure S5

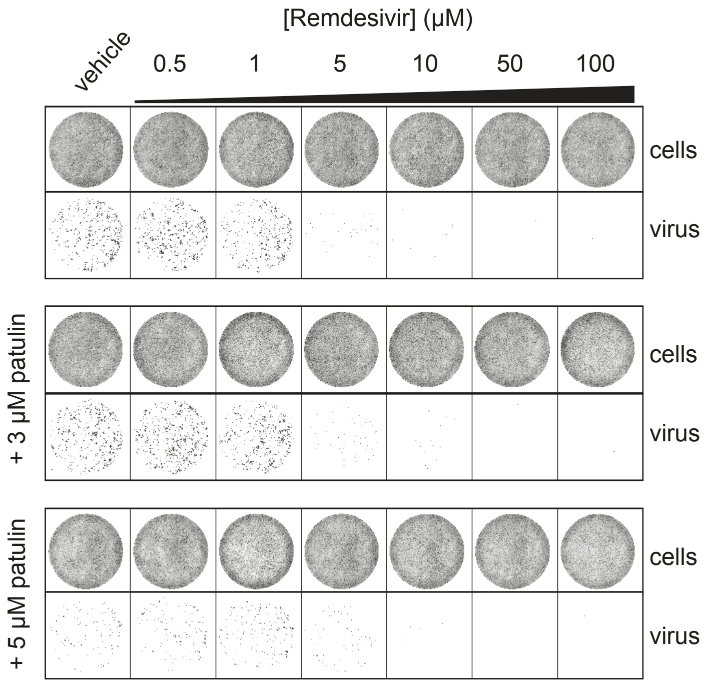

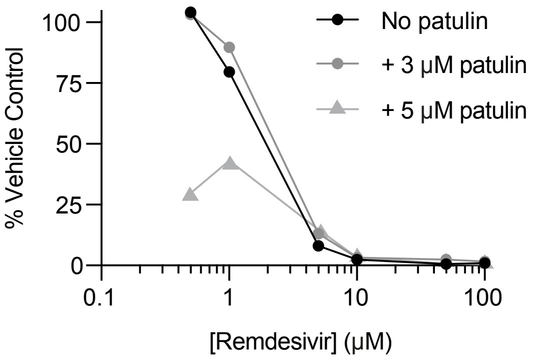

Supplementary Table S1. Constructs and Purification Strategies

Construct name	Used to express	Codon optimisation	Purification steps	Construct Id	Cloning sites o oligos	Antibiotic resistance
pET11a_3xFlag-nsp10	Individual nsp10	*E. coli*	Pull down (PD) α-Flag > MonoQ > Dialysis	Addgene 169157	NdeI, BamHI	Amp
pK27SUMO_14His-SUMO-nsp14	Individual nsp14	Non-optimised	PD NiNTA > Ulp1 > Superdex200	MRC PPU DU70487	3, 4	Kan
pCOLADuet-1_nsp10	Complex nsp14/10	*E. coli*	PD α-Flag > MonoQ > Superdex200	Addgene 169158	NcoI, BamHI	Kan
pET11a_3xFlag-nsp14		*E. coli*		Addgene 169159	NdeI, BamHI	Amp
pK27SUMO_14His-SUMO-nsp14-GGSGGS-nsp10	nsp14-10 fusion	*E. coli*	PD NiNTA > Ulp1 > MonoQ > Superdex200	Addgene 169160	3, 4	Kan
pK27SUMO_14His-SUMO-nsp10-GGSGGS-nsp14	nsp10-14 fusion	*E. coli*	PD NiNTA > Ulp1 > MonoQ > Superdex200	Addgene 169161	3, 4	Kan
pBIG1b_nsp10-6His-3xFlag	Individual nsp10	Insect cells	PD α-Flag > MonoQ > Dialysis	Addgene 169162	BamHI, HindIII	Amp + Spectin.
pBIG1a_3xFlag-6His-nsp14	Individual nsp14	Insect cells	PD α-Flag > Superdex200	Addgene 169163	BamHI, HindIII	Amp + Spectin.
pBIG2ab_nsp14/nsp10-6His-3xFlag	Complex nsp14/10	Insect cells	PD α-Flag > MonoQ > Superdex200	Addgene 169164	PmeI	Amp. + Chlor.

Supplementary Table S2. Cloning oligonucleotides	
**Oligo**	**Oligo sequence**
1	GCCACCAATCTGTTCTCTGTG
2	TAATAAgcggccgcacatcac

Supplementary Table S3. Substrate oligonucleotides		
**Oligo**	**Substrate**	**Oligo sequence (5' → 3')**
i	Top unlabelled RNA	AAUAGCUUCUUAGGAGAAUGAC
ii	Top Cy5 RNA	Cy5-AAUAGCUUCUUAGGAGAAUGAC
iii	Bottom unlabelled RNA	CCCUAUCGUCAUUCUCCUAAGA
iv	Top Cy3 RNA	Cy3-GGUAGUAAUCCGCUC
v	Bottom quencher RNA	UUUUUUUUUUUUUUUUUUUUGAGCGGAUUACUACC-Iowa Black RQ
vi	Top Cy3 **DNA**	Cy3-GGTAGTAATCCGCTC
vii	Bottom quencher DNA	TTTTTTTTTTTTTTTTTTTTGAGCGGATTACTACC-Iowa Black RQ

Supplementary Table S4. Top Screen Hit Compounds						
**Chemical Name**	**Cat No**	**Aggreg. Index (LogP)**	**Residual activity (end-point screen)**	**Z score (end-point screen)**	**Residual activity (kinetic screen)**	**Z score (kinetic screen)**
Patulin	NP-223	-0.4	0.89	-1.30	0.70	-4.81
Mercaptopurine	S1305	-0.4	0.96	-0.54	1.00	0.27
Aurintricarboxylic acid (ATA)	A 1895	3.8	-1.85	-31.00	0.66	-5.47
1,3-PBIT dihydrobromide (PBIT)	P 8227	1.5	0.94	-0.77	0.82	-2.77
Coptisine chloride	HY-N0736	0.2	0.87	-1.55	0.81	-2.82
Betaxolol hydrochloride	B 5683	2.8	0.90	-1.21	0.86	-2.12
Bisdemethoxycurcumin (Curcumin III)	HY-N0007	2.7 (80% similarity to aggregator)	-0.08	-11.80	0.88	-1.70
Demethyl-curcumin	MFCD03427310	2.5 (100% similarity to aggregator)	0.37	-6.94	0.93	-0.84
JNJ-1661010	S2828	3.9	0.63	-4.17	0.87	-1.82
Phenazine methosulfate	MFCD00011923	-0.4	0.83	-1.91	0.94	-0.74
Betahistine dihydrochloride	S3176	0.7	1.03	0.22	0.86	-1.97
Valinomycin	KC-140	5.1	0.98	-0.34	0.83	-2.61
